# Improving care quality and preventing maltreatment in institutional care – a feasibility study with caregivers

**DOI:** 10.3389/fpsyg.2015.00937

**Published:** 2015-07-14

**Authors:** Katharin Hermenau, Elisa Kaltenbach, Getrude Mkinga, Tobias Hecker

**Affiliations:** ^1^Department of Psychology, University of Konstanz, KonstanzGermany; ^2^vivo internationalwww.vivo.orgKonstanz, Germany; ^3^Department of Educational Psychology, University of Dar es SalaamDar es Salaam, Tanzania; ^4^Department of Psychology, University of ZurichZurich, Switzerland

**Keywords:** child maltreatment, care quality, violence, institutional care, mental health, orphans, attachment, Sub-Saharan Africa

## Abstract

Institutionalized children in low-income countries often face maltreatment and inadequate caregiving. In addition to prior traumatization and other childhood adversities in the family of origin, abuse and neglect in institutional care are linked to various mental health problems. By providing a manualized training workshop for caregivers, we aimed at improving care quality and preventing maltreatment in institutional care. In *Study 1*, 29 participating caregivers rated feasibility and efficacy of the training immediately before, directly after, and 3 months following the training workshop. The results showed high demand, good feasibility, high motivation, and acceptance of caregivers. They reported improvements in caregiver–child relationships, as well as in the children’s behavior. *Study 2* assessed exposure to maltreatment and the mental health of 28 orphans living in one institution in which all caregivers had been trained. The children were interviewed 20 months before, 1 month before, and 3 months after the training. Children reported a decrease in physical maltreatment and assessments showed a decrease in mental health problems. Our approach seems feasible under challenging circumstances and provides first hints for its efficacy. These promising findings call for further studies testing the efficacy and sustainability of this maltreatment prevention approach.

## Introduction

In many low-income countries, particularly in Sub-Saharan Africa and South Asia, the number of orphans has been constantly rising over the last decades with estimated numbers of 56,000,000 and 40,800,000 respectively, for example as a consequence of poverty, the HIV/AIDS epidemic or political conflicts (Maundeni and Malinga-Musamba, 2013; United Nations Children’s Fund, 2014). These countries are often overburdened with the high number of orphans; and family or community-based care cannot be provided for all children in need (Li et al., 2008). Thus, it is not surprising that child care institutions like orphanages and children’s homes still constitute the most frequently applied way of caring for children without parents (Neimetz, 2010; Rygaard, 2010). However, caregiving quality in institutional care seems to be deficient in most cases (Bakermans-Kranenburg et al., 2008). The caregiver–child ratio is often high, with mainly perfunctory and insensitive interactions (Muhamedrahimov et al., 2004; van Ijzendoorn et al., 2011). High staff turnover, no assignment of caregivers to specific children, and children’s frequent changes of institution additionally illustrate the instability and inconsistency of the children’s environment in institutional care (Muhamedrahimov et al., 2004; Wright et al., 2014). As a consequence many children in institutional care are at high risk to experience neglect. It occurs most typically in the form of socio-emotional neglect (Bakermans-Kranenburg et al., 2008; The St. Petersburg-USA Orphanage Research Team, 2008). For example, in a survey of orphanages in Kazakhstan, more than one fifth of the children reported being severely neglected (Haarr, 2011). However, in addition to various childhood adversities in the family of origin and deficient caregiving and neglect in institutional care, orphans often experience further maltreatment in institutional care (Pinheiro, 2006). Unfortunately, exposure to violence and other forms of abuse in institutional care have received comparably little attention, although many children worldwide frequently face physical, emotional, and sexual violence (UNICEF, 2014). For example, in a national, representative survey in Tanzania, the majority of adolescents (almost 75%) reported exposure to physical violence, one quarter to emotional maltreatment, and 28% of the girls and 13% of the boys to sexual violence (UNICEF, 2011). Harsh discipline methods like corporal punishment (e.g., spanking or beating with objects) and emotional punishment (e.g., insulting, humiliating) are highly prevalent in many countries worldwide (Straus, 2010; UNICEF, 2014). UNICEF (2014) reported that more than seven in 10 children between 2 and 14 years of age in Sub-Saharan Africa, the Middle East, and North Africa experience violent disciplining. The already high rates of maltreatment experienced by children in the general population are estimated to be even higher in institutional care (Euser et al., 2014; UNICEF, 2014). Gavrilovici (2014) found over 80% of the children reporting physical violence in Romanian orphanages. Similar results with maltreatment experienced by most of the children were found in studies in orphanages in Tanzania (Hermenau et al., 2011, 2014).

Institutionalized children often face various adversities early in their lives, such as parental loss or maltreatment in the family of origin with detrimental consequences for their healthy development (Li et al., 2008). Institutionalization, with its depriving environment, no stable attachment figures, and further maltreatment, may have an additional negative impact on the children (MacLean, 2003; Johnson et al., 2006; Hermenau et al., 2011). Accordingly, higher rates of several psychological problems, for instance symptoms of depression and post-traumatic stress disorder, were reported for institutionalized children (Zeanah et al., 2006; Hermenau et al., 2011). They are also at heightened risk to develop externalizing problems, such as aggressive behavior or attention deficit hyperactivity disorder, compared to community samples (Erol et al., 2010; EL Koumi et al., 2012). Furthermore, researchers found developmental delays in all areas (MacLean, 2003; Johnson et al., 2006; Hearst et al., 2014). So far, the long-term impact of institutionalization has only been marginally studied. Sigal et al. (2003) found more mental health problems, chronic diseases, and social isolation in middle-aged adults who were institutionalized as children, indicating that institutionalization may also have serious long-term consequences. Furthermore, studies have revealed that maltreatment by caregivers in institutional care is positively correlated with mental health problems, especially in children who were institutionalized at young age (Hermenau et al., 2011, 2014). These results are supported by general findings indicating that maltreatment has a negative impact on children’s mental and physical health (Gershoff, 2002, 2013; Hecker et al., 2014).

So far, little is known about the caregivers that work in institutional care in low-income countries. The few existing studies indicate that these caregivers rarely have specialized qualification in child care and face extremely poor working conditions (Heron and Chakrabarti, 2002; Muhamedrahimov et al., 2004; Rygaard, 2010). Generally, they report a high workload and stress level (Vashchenko et al., 2010; Wright et al., 2014). Muhamedrahimov et al. (2004) found higher depression and anxiety levels in caregivers compared to a community sample. Thus far, no study researched the impact of caregivers’ stress levels on the mental health of institutionalized children. However, parental stress is known to affect child development, well-being, and the parent–child relationship (Mäntymaa et al., 2012; Widarsson et al., 2013). Therefore, it would not be surprising if enhanced stress levels of caregivers may have similar effects.

All in all, the detrimental consequences of institutionalization for children’s well-being, and the current difficulties to provide family or community-based care alternatives, particularly in resource-poor countries, show the necessity to improve institutional care (Rygaard, 2010; McCall, 2013). A well-studied example is the St. Petersburg study (Groark et al., 2005) showing positive effects for combined caregiver training with structural changes up to 6 years follow-up (McCall et al., 2013). Most of the current intervention studies focus solely on improving care quality and preventing neglect. Despite the high risk of maltreatment in institutional care, interventions that focus additionally on the prevention of violence and abuse are rare. Hermenau et al. (2011) addressed this issue and conducted an intervention in a Tanzanian orphanage by training caregivers in non-violent parenting alternatives and introducing a ban of violent punishment. As a result children reported a decline of exposure to violent punishment. Overall, intervention studies reported improvements in caregiving quality, children’s mental health, and development (Bakermans-Kranenburg et al., 2008; Hermenau et al., 2011; Berument, 2013). Additionally, caregivers reported less work-related stress, depression, anxiety symptoms, and more social behavior toward the children was observed (Taneja et al., 2002; The St. Petersburg-USA Orphanage Research Team, 2008).

Interventions aiming to improve care quality in institutional care have shown promising results. Nevertheless, interventions focusing on both improving care quality and preventing maltreatment are scarce. To close this gap, we conducted a feasibility study focusing additionally on maltreatment prevention. In Study 1, we evaluated the feasibility and implementation of the training, as well as the success from the caregivers’ perspective. We hypothesized that our approach would be feasible to conduct and that the caregivers would demand such training and be motivated to participate. First evidence for the efficacy of the training will be examined. Parallel in time, Study 2 focused on the orphans’ experiences of physical and emotional maltreatment as well as their mental health before and after their caregivers had participated in the training workshop. The training workshop took place between pre-assessment (t1) and follow-up assessment (t3). We hypothesized that the children’s exposure to maltreatment by their caregivers would decline after the training with positive effects on the children’s mental health.

## Study 1

### Materials and Methods

#### Sample

Twenty-nine caregivers attended a 2-weeks training. The caregivers worked in different institutions in a town of ∼150,000 inhabitants in Southern Tanzania. In total, 54% (*n* = 15 of 28) worked as caregivers in school projects for orphans between 3 and 16 years and 46% (*n* = 13 of 28) in orphanages or other institutional care facilities for children between 0 and 15 years. On average they had worked 25.93 months (SD = 30.14, range 1.5–105.5, *n* = 28) at their current place of work. The caregivers reported that housekeeping activities took up 7.12 h (SD = 3.88, range 0–14, *n* = 26) per day, while only 2.88 h (SD = 2.67, range 1–9, *n* = 17) were spent on the interaction with children. The average caregiver–child ratio was 1:20. The vast majority of caregivers were female (90%, *n* = 26 of 29). Age range was 19–76 years, with an average age of 36.72 years (SD = 12.85, *n* = 28). Trainees reported on average 7.27 years of formal education (SD = 2.38, range 3–12, *n* = 28). None of them had undergone any training in child care. Most of the trainees reported exposure to maltreatment in their childhood (88%, *n* = 14 of 16). Additionally, 87% (*n* = 13 of 15) reported the frequent use of physical punishment in their daily work.

#### Design and Procedure

**FIGURE 1:**
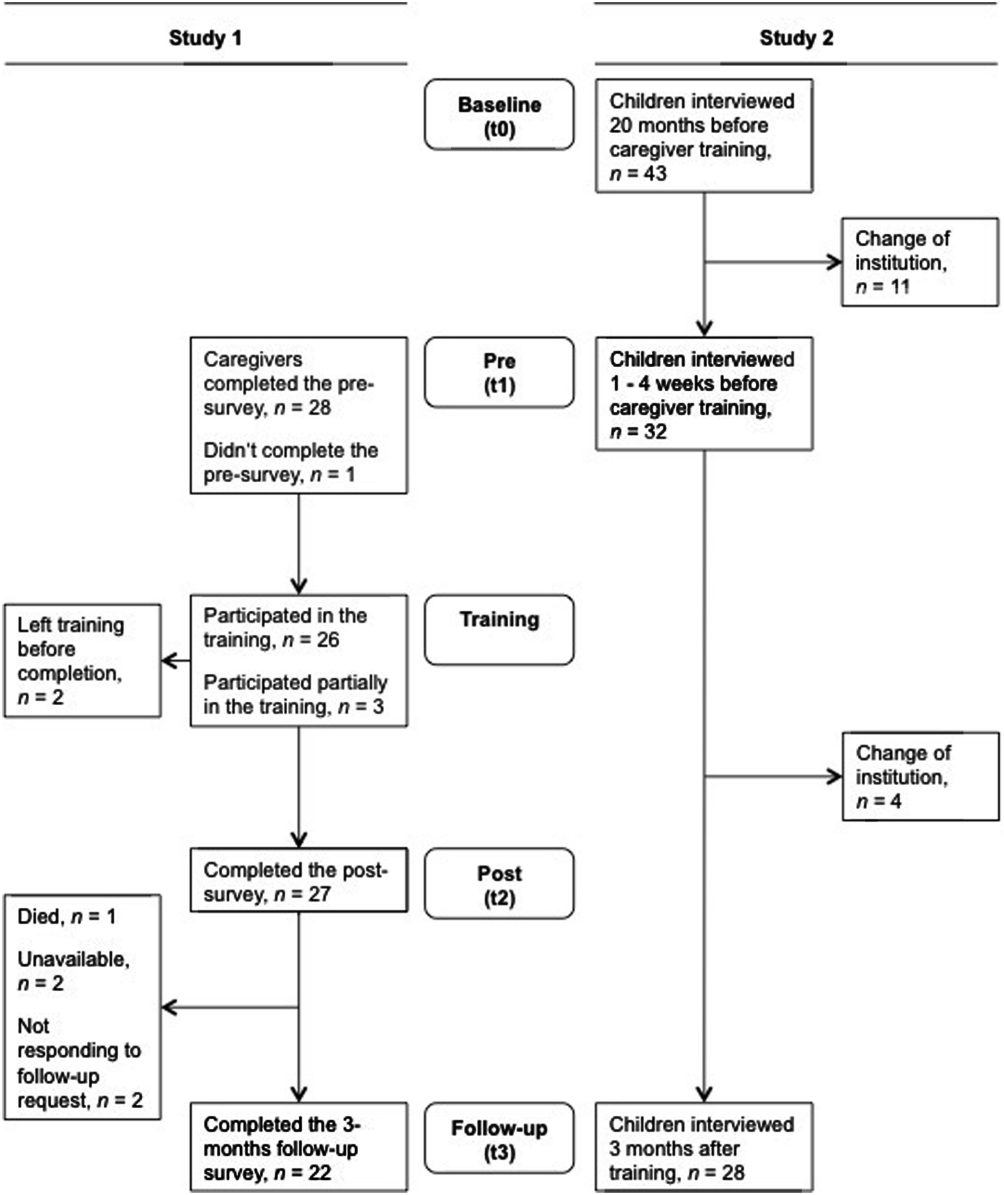
**Flow chart of the study design**.

The caregiver training was held on the premises of a primary school and lasted 2 weeks, taking place 8 h per day, 6 days per week. All caregivers participated voluntarily, however, the management of respective child care institutions showed a great interest in the workshop training. In some cases this may have resulted in some pressure to join the training workshop. Participants were assured that all information given during the training would be kept strictly confidential and that their employers would receive no individual information from the research team. No participation fee was charged and no financial compensation was given to the trainees but food and beverages were provided. Data was collected from the caregivers prior to, shortly after, and 3 months following the training. The training was conducted during school holiday time. Nevertheless some caregivers could not participate fully in the training because of the necessity to care for the children staying in the respective institutions (10%, *n* = 3 of 29). Correspondingly the number of completed surveys at the different time points varied (for detailed information see **Figure 1**). Drop-out at follow-up was 24% (*n* = 7 of 29).

One Tanzanian (GM) and one German (TH) psychologist conducted the training workshop. Both had extensive work experience in caregiving and maltreatment prevention. Two psychologists and a nurse from Germany supported the trainer team. Three trained Tanzanian interpreters facilitated communication. The study was approved by the Tanzanian Commission for Science and Technology and the Ethical Review Board of the University of Konstanz.

#### Intervention

The intervention consisted of a 2-weeks training workshop for caregivers working in institutional care. The training concept was based on the parenting guidelines of The American Academy of Pediatrics (1999) and the FairstartGlobal training concept (Rygaard, 2010).

Key principles of the training workshop were: (a) Participative approach: trainees were invited to participate actively, to tailor the training, and to develop their own strategies on how to implement the training content in their daily work (Wright et al., 2014). (b) Practicing: lectures were followed by units of practice in order to enable the trainees to use the acquired skills in real life. (c) Trustful atmosphere: trainees were encouraged to talk openly about work problems and their own experiences of maltreatment, with the aim of creating a trusting and open atmosphere assuring confidentiality (Rygaard, 2010). (d) Sustainability: sustainability of the training was ensured through intensive practicing, repetition of the new knowledge, self-reflection, and the training component *team work and supervision* described below (Sparling et al., 2005; McCall et al., 2013).

The training was based on seven core components, conducted in the order specified beneath:

##### Child development

Trainees discussed the needs of children and theoretical input was given about child development. Subsequently, trainees practiced forming age-appropriate expectations and caring-approaches. The aim of this training section was to foster empathy and understanding toward the children and to enable the trainees to better assess the children’s abilities and thus to form age-appropriate expectations (McCall et al., 2010; Hermenau et al., 2011).

##### Caregiver–child relationship

The importance of secure attachment and bonding for children was emphasized. Trainees elaborated on the implications of being a parenting figure and role model. Basic communication skills in giving good instructions and communicating expectations were presented and practiced. This section aimed to point out the importance of secure attachment and bonding as well as elements of how to establish and improve a caregiver–child relationship (Groark et al., 2005; Rygaard, 2010).

##### Effective caregiving strategies

Different strategies to maintain good behavior and to change misbehavior (e.g., reinforcement systems, privilege removal, contracts) were introduced and subsequently practiced in small groups using interactive elements such as role plays. The aim was to provide alternative caregiving strategies in place of harmful discipline measures and to reduce feelings of helplessness (Hermenau et al., 2011).

##### Maltreatment prevention

Trainees reflected on their own experiences of maltreatment during childhood and their own use of harsh punishment toward children. Common caregiving and discipline strategies in Tanzania were discussed, consequences of harmful punishment were pointed out, and myths about physical punishment were examined. The aim was to raise awareness toward the detrimental consequences of maltreatment. This section was closely linked with the newly learned effective caregiving strategies in order to show alternatives to violent punishment (Dubanoski et al., 1983; Hermenau et al., 2011).

##### Supporting burdened children

This component aimed at providing knowledge about common emotional and behavioral problems children face, such as stress, depression, oppositional behavior, bedwetting, delayed development, and being HIV positive, as well as introducing strategies for handling such challenges (Hermenau et al., 2011).

##### Child-centered institutional care

Although structural changes were not implemented in this intervention, the importance of an adequate caregiver–child ratio, stable caregivers, and family-like groups was elucidated. In small groups trainees discussed ideas and created plans how to introduce changes to their particular workplace. Further, aspects like safety, the importance of play for a healthy development, structures, rituals, and rules, were introduced and subsequently discussed in detail. The aim of this section was to enable the trainees to realize changes that are possible at their specific workplace in order to improve the children’s and caregivers’ conditions (Groark et al., 2005; Sparling et al., 2005).

##### Team work and supervision

The importance of a good work atmosphere and supporting colleagues was discussed. Possibilities for supervision and where to seek help were presented. This component aimed at improving the immediate working conditions and ensuring the implementation of the training contents in the workplace (Sparling et al., 2005; Rygaard, 2010; McCall, 2013).

#### Measures

Trainees filled out surveys at three different time points (see supplementary files). The questions were translated word-by-word from English into Swahili and the Swahili version was discussed extensively to ensure a precise translation. Major parts of the surveys were purpose-built for this training since no suitable questionnaires were available. One part of the questions was qualitative to gather information in the context of this pilot study (e.g., *information about the workplace, experience as a caregiver, daily tasks*). Qualitative information was used to describe the context of this study. For this purpose, information was classified into pre-defined categories by two independent raters. Prior to the training (t1), sociodemographic information (e.g., *age, sex, education*), the demand for training (e.g., *own exposure to child maltreatment, use of physical punishment, current work load*), and the motivation to participate were assessed. Further, caregivers’ stress levels were assessed with the work-related burnout section of the Copenhagen Burnout Inventory (CBI; Kristensen et al., 2005). Sum scores ranging from 0 to 100 were computed. Low burnout was defined as <50 and high burnout as >50 (Borritz et al., 2006). The feasibility of the training was measured during the training by the trainers (e.g., *time management, participation, trainees’ comprehension of the topic, trainees’ motivation, and overall feasibility*) and after the training (t2) by the trainees’ satisfaction with the training (e.g., *How satisfied were you with the training? Would you recommend this workshop to other caregivers?*). After the training (t2), efficacy measures were assessed; including questions about the trainees’ changes in attitude (e.g., *How did the workshop influence your understanding of the children?*), their motivation to change their caregiving behavior (e.g., *How many aspects of the workshop can you use in your daily work?*), and an exam to monitor understanding and theoretical knowledge of the training content (see supplementary files). At the 3 months follow-up (t3) further efficacy measures, about the integration of the learned content into daily work (e.g., *Which aspects of the workshop do you use in your daily work?*) and the success of the training from the caregivers’ perspective were assessed (e.g., *Did the relation to the children you care for change because of the workshop?, Do you see a change in the behavior of the children you care for because you are acting differently*). For detailed information about the surveys, see supplementary files.

#### Data Analysis

To report qualitative data, categories were generated by clustering the answers. The formed categories and the allocated answers were double-checked by independent raters. Multiple responses were possible; correspondingly total percentage scores were higher than 100%. All analyses were performed with SPSS, version 21.

### Results

#### Demand

Beside the fact that none of the trainees had undergone any training in child care and most of the trainees reported both exposure to maltreatment in their childhood and the frequent use of physical punishment in their daily work (see above), they also reported a very high workload. At t1 the trainees rated their current workload on a scale from 0 (*low workload*) to 10 (*high workload*) with *M* = 7.22 (SD = 2.89, range 0–10, *n* = 27). Working hours per week were on average 73.74 h (SD = 42.98, range 36–168, *n* = 26). Concordantly, 33% (*n* = 9 of 27) of the trainees showed high burnout symptoms on the CBI scale (*M* = 38.36, SD = 12.98, range 17.86–64.29, *n* = 27).

#### Motivation

Trainees rated their motivation to participate in the training as very high (*M* = 9.96, SD = 0.20, range 9–10, *n* = 26; scale 0 = *not at all* to 10 = *very much*). Coherent with this, they expected the training to help them a lot in their daily work (*M* = 10.00, SD = 0.00, range 10–10, *n* = 26; scale 0 = *not at all* to 10 = *very much*).

#### Feasibility of the training

The trainers evaluated each training session (*N* = 43) on a four-point Likert scale (0 = *unsatisfying* to 3 = *excellent*) regarding participation, comprehension, motivation, time management, and feasibility. Scores were then added to a sum score ranking from 0 to 15. The sum score indicated a high contentment of the trainers (*M* = 9.14, SD = 3.55, range 2–15, *N* = 43). Moreover, the trainees reported high satisfaction with the training at t2 (*M* = 2.81, SD = 0.57, range 1–3, *n* = 26; scale 0 = *unsatisfying* to 3 = *excellent*). All trainees (100%, *n* = 27) indicated that they would recommend the training to others and 89% (*n* = 24 of 27) stated that they would be willing to contribute money for participating in the training.

#### Efficacy

At t2 all trainees (100%, *n* = 27) reported a better understanding of the children they care for. The majority of the trainees (88%, *n* = 21 of 24) anticipated that by implementing the training aspects into their daily work, their workload would be lower, while 13% (*n* = 3 of 24) assumed their workload would increase. Additionally, in the exam subsequent to the training, trainees answered 77% (SD = 15%, range 47–100%, *n* = 27) of the questions on the training content correctly. According to the Tanzanian school system, 22% (*n* = 6 of 27) showed an *excellent*, 22% (*n* = 6 of 27) a *good,* 22% (*n* = 6 of 27) a *fully satisfactory,* and 26% (*n* = 7 of 27) a *satisfactory* performance in the exam. Only, two trainees (with 47 and 48%) failed to achieve the required 50% to pass the exam. The written exam was highly challenging for some of our trainees as they had only relatively low reading and writing skills. All in all, the results of the exam indicate that trainees understood the instructions in the training and were able to reproduce them satisfactorily in the exam.

At t3, the amount of the training aspects used in daily work was rated on a five-point Likert scale, with *M* = 2.55 (SD = 1.10, range 1–4; *n* = 22; scale 0 = *no aspects* to 4 = *all aspects*), showing a high use of the training aspects. In detail, 50% (*n* = 11 of 22) reported using aspects of alternative caregiving strategies, 36% (*n* = 8 of 22) of supporting burdened children, and 36% (*n* = 8 of 22) of child-centered institutional care. Furthermore, the caregivers reported a better relationship to the children (*M* = 3.36, SD = 0.49, range 3–4, *n* = 22; scale 0 = *much worse* to 4 = *much better*) and a positive change in the behavior of the children as a result of their own modified behavior (*M* = 3.27, SD = 0.55, range 2–4, *n* = 22; scale 0 = *much worse* to 4 = *much better*). **Table 1** summarizes the main results of Study 1.

**Table 1:** Summary of the main results (Study 1).

Categories	Results
Demand	• No caregiver had undergone a training in childcare (t1) • Trainees (88%) reported own exposure to childhood maltreatment (t1) • Trainees (87%) reported the frequent use of physical punishment (t1)
Motivation	• High motivation to participate (t1) • High expectation concerning the usefulness of the training (t1)
Feasibility of the training	• High contentment of the trainers (t2) • High satisfaction of the trainees (t2) • All trainees recommended the training (t2)
Efficacy	• Trainees reported frequent use of training content in daily work (t3) • Trainees (100%) reported a better relationship to the children (t3) • Trainees (96%) reported a positive change in children’s behavior (t3)

*t1, pre-assessment, directly before intervention; t2, post-assessment, directly after intervention; t3, follow-up assessment, 3 months after intervention*.

## Study 2

### Materials and Methods

#### Sample

Children from one orphanage at which all caregivers participated in the training workshop introduced in Study 1 were interviewed. Due to children’s frequent changes of institution, we only included *N* = 28 children (of a total of 50) who had lived in the orphanage at all survey time points. Fifty percent (*n* = 14) of the trainees were female. The average age at the baseline interview was 9.79 years (SD = 1.45, range 7–12). Most of the children were full orphans (93%, *n* = 26), two children (7%) had lost one parent.

#### Design and Procedure

The children were interviewed at three time points to measure changes in their exposure to maltreatment by caregivers and in their mental health before and after the intervention. Baseline interviews (t0) were conducted 20 months before the intervention within the scope of another study, in which 409 primary school students were interviewed, 43 of them living in the assigned orphanage (Hecker et al., 2014; Hermenau et al., 2014). The high number of children, particularly orphans, who suffered from mental health problems and poor school performance have led to the employment of a counseling psychologist. The pre-assessment (t1) was conducted 1–4 weeks before the intervention with 32 children. Three months after the intervention, a follow-up assessment (t3) with 28 children took place (see **Figure 1** for further information on the study design). The drop-out rate was 35% (*n* = 15 of 43) due to the high rate of children changing institutions. The study was approved by the Tanzanian Commission for Science and Technology and the Ethical Review Board of the University of Konstanz. Baseline interviews were conducted by a team of German and Tanzanian psychologists, partially with trained interpreters. A Tanzanian psychologist conducted the pre- and follow-up surveys. All interviewers were trained intensively in the concepts of mental disorders and interview techniques. Each child was interviewed in a calm and private setting. The interview at the baseline survey took ∼1.5 h, the pre-survey 1 h, and the follow-up survey 30 min.

#### Measures

All instruments were applied as a structured interview in Swahili. The translated mental health instruments were already successfully used in a comparable sample of orphans in Tanzania (Hermenau et al., 2011). New questions were translated word-by-word from English into Swahili and the Swahili version was discussed intensively to ensure a correct and valid translation. The first part of the interview consisted of sociodemographic questions, assessing age and sex.

Children’s reports of physical and emotional maltreatment by the caregivers in the orphanage were assessed at different time points. Physical maltreatment was defined as being spanked or beaten and emotional maltreatment was defined as being yelled or screamed at. At t0, adverse childhood experiences were assessed with the Maltreatment and Abuse Chronology of Exposure – Pediatric Interview which is the child-appropriate version of the Maltreatment and Abuse Chronology of Exposure (Teicher and Parigger, 2015; Isele et al., submitted). As the present study focused on the children’s report regarding physical and emotional maltreatment from the caregivers, only a part of the items was used. Accordingly, at t1 and t3, maltreatment was assessed in concise form. Additionally, an open question about caregivers’ ways of punishment (*If you do something wrong how do your caregivers react?*) was asked at t1 and t3. Two independent raters classified the answers into the following pre-defined categories: *physical maltreatment, emotional maltreatment*, and *explanation (i.e., caregiver explains the child what he/she did wrong)*. There was no case of discrepancy between the two independent raters.

The Children’s Depression Inventory (CDI) is a widely used self-report instrument to measure depressive symptoms in children and adolescents (Sitarenios and Kovacs, 1999; Kovacs, 2001). Validity and reliability are high (Verhulst and van der Ende, 2006) and the CDI has been implemented successfully in Tanzania (Traube et al., 2010; Hermenau et al., 2011). It is originally a self-report measure consisting of 27 items in which the child chooses the best fitting statement out of three possible alternatives. The overall score which can be calculated by adding all item scores ranges from 0 to 54, with higher values indicating more severe depressive symptoms. Cronbach’s α coefficient was 0.60 for the present baseline sample (*N* = 28) and Cohen’s κ coefficient measuring the interrater reliability was 0.99 (range 0.92–1) for the entire baseline sample (*N* = 409).

Self-reported internalizing and externalizing problems of children were measured with the Strengths and Difficulties Questionnaire (SDQ; Goodman et al., 1998). It is known as an effective instrument for brief behavior screenings and shows good reliability and validity, also in international contexts (Woerner et al., 2004; Hermenau et al., 2011). We used the self-report version for children between 11 and 16 years in interview form. Previous research has shown that this version can also be used with children from the age of eight and onward (Muris et al., 2004). The SDQ consists of 25 items rated on a three-point Likert scale. In this study, a total difficulties score was calculated by adding all item scores, excluding five items regarding prosocial behavior. The total score ranges from 0 to 40, with higher scores indicating more severe internalizing and externalizing problems. Cronbach’s α coefficient was 0.60 for the present baseline sample and Cohen’s κ coefficient was 0.99 (range 0.94–1) for the entire baseline sample.

With the Reactive-Proactive Questionnaire (RPQ) we assessed self-reported aggressive behavior of the children (Raine et al., 2006). Psychometric measures are good (Cima et al., 2013) and the instrument has been internationally used (Fung et al., 2009). It consists of 23 items which are rated on a three-point Likert scale. The questionnaire was adjusted to the conditions in Tanzania following Hermenau et al. (2011): one item was removed (Item 18: *How often have you made obscene phone calls for fun?*) and two items were slightly changed (Item 4: *children* instead of *students*; Item 9: *fight* instead of *gang fight*). A total aggression score ranging from 0 to 44 was calculated, with higher scores indicating more aggressive behavior. Cronbach’s α coefficient was 0.89 for the present baseline sample and Cohen’s κ coefficient was 0.99 (range 0.94–1) for the entire baseline sample.

#### Data Analysis

All measured variables met the preconditions for parametric analysis as defined by West et al. (1995). No outliers were detected. One-way repeated-measures analyses of variance (ANOVA) were calculated to measure changes of the mental health sum scores over time. Assumption of sphericity, tested with the Mauchly’s test, was met for all ANOVAs. Bakeman (2005) recommends generalized eta squared ($\eta_{g}^{2}$) as effect size for repeated-measures ANOVAs because of its comparability across study designs (Olejnik and Algina, 2003). Generalized eta squared can be classified into small (0.02), medium (0.13), and large (0.26) effect sizes (Bakeman, 2005). Additionally, paired *t*-tests with Cohen’s *d* as effect size were calculated as *post hoc* tests. According to Cohen (1992), 0.20 indicates a small effect, 0.50 a medium effect, and 0.80 a large effect. Since the exposure to maltreatment was coded as a dichotomous variable, Cochran’s *Q* was used for calculations with three time points and McNemar for calculation with two time points and as *post hoc* test of Cochran’s *Q*. Discordant pairs were less than 25, therefore exact probabilities based on the cumulative binomial distribution are provided for McNemar calculations. As an effect size for McNemar, phi (φ) will be reported, with 0.10 defining a small, 0.30 a medium, and 0.50 a large effect. To prevent alpha-inflation, Bonferroni–Holm correction was used for all multiple comparisons. We used an α-level of 0.05 to compare t1 and t3, an adjusted α-level of 0.025 for t0 with t3, and 0.017 for t0 with t1. As hypotheses were directional, analyses were calculated one-tailed. All analyses were performed with SPSS, version 21 for Windows.

### Results

#### Exposure to Maltreatment

**FIGURE 2:**
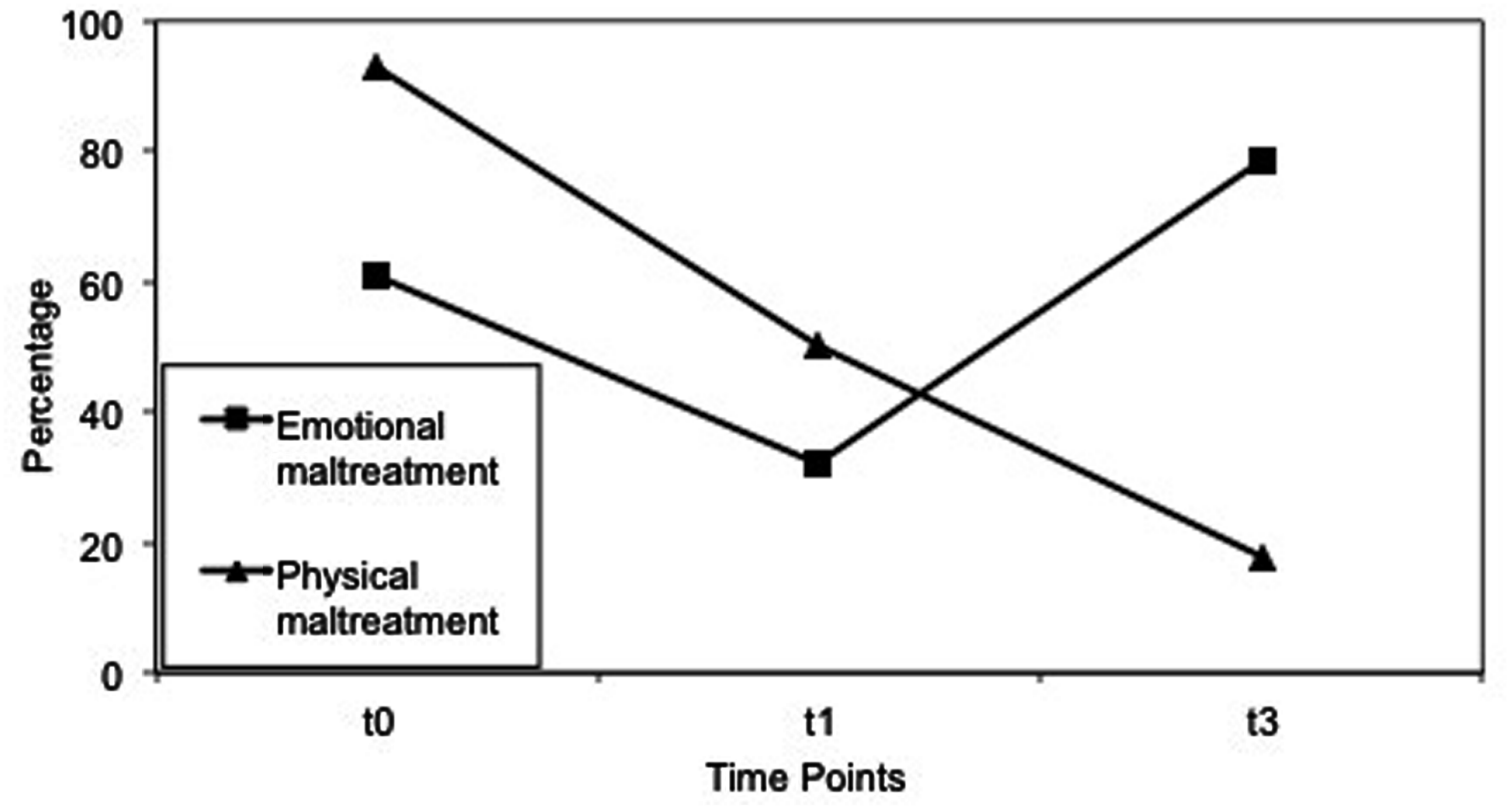
**Physical and emotional maltreatment reported by the children at t0, t1, t3. t0, baseline assessment, 20 months before intervention; t1, pre-assessment, 1–4 weeks before intervention; t3, follow-up assessment, 3 months after intervention**.

Exposure to physical and emotional maltreatment by caregivers was analyzed regarding changes between the survey time points (**Figure 2**). Ninety-three percent of the children reported physical maltreatment at t0, 50% at t1, and 18% at t3. Cochran’s *Q* revealed a significant change in physical maltreatment over the three time points, *Q*(2) = 25.62, *p* < 0.001, *N* = 28. Pairwise comparisons between the time points with McNemar showed significantly less physical maltreatment at t3 compared to t1, χ^2^(1) = 4.27, *p* = 0.018, φ = 0.39, and to t0, χ^2^(1) = 17.39, *p* < 0.001, φ = 0.79. Additionally, physical maltreatment rates at t1 compared to t0 were lower, χ^2^(1) = 8.64, *p* = 0.001, φ = 0.56. Emotional maltreatment was reported by 61% of the children at t0, 32% at t1, and 79% at t3. Cochran’s *Q* showed no significant decrease across the survey time points. Children’s responses to the open question about caregivers’ ways of punishment differed between t1 and t3: less physical maltreatment was named at t3 (28%, *n* = 6) compared to t1 (64%, *n* = 18), χ^2^(1) = 6.72, *p* = 0.004, *N* = 28, φ = 0.49. No change in child-reported emotional maltreatment was found, χ^2^ (1) = 0.00, *p* = 0.500, *N* = 28, φ < 0.01. At t3 children reported more often (57%, *n* = 16) than at t1 (29%, *n* = 8) that caregivers explained to them what they did wrong, χ^2^(1) = 3.06, *p* = 0.038, *N* = 28, φ = 0.33.

#### Mental Health

Repeated-measures ANOVAs were performed to capture changes in the severity of depressive symptoms, internalizing and externalizing problems, and aggressive behavior over time (**Table 2**). Depressive symptoms changed significantly over time with a large effect size, *F*(2,52) = 15.00, *p* < 0.001, *n* = 27, $\eta_{g}^{2}$ = 0.31. *Post hoc t*-tests revealed a significant decrease of CDI scores between t1 and t3, *t*(26) = 4.99, *p* < 0.001, *d* = 0.96, as well as between t0 and t3, *t*(26) = 4.76, *p* < 0.001, *d* = 0.92. No significant difference was found between t0 and t1, *t*(26) = 0.87, *p* = 0.196, *d* = 0.17. Internalizing and externalizing problems also changed significantly over time, *F*(2,54) = 12.58, *p* < 0.001, *N* = 28, $\eta_{g}^{2}$ = 0.28. *Post hoc t*-tests showed lower SDQ scores at t3 in comparison to t1, *t*(27) = 1.80, *p* = 0.042, *d* = 0.34, and to t0, *t*(27) = 4.67, *p* < 0.001, *d* = 0.88. Additionally, internalizing and externalizing problems were lower at t1 than at t0, *t*(27) = 3.14, *p* = 0.002, *d* = 0.59. Similar results were found for aggressive behavior: RPQ scores significantly changed over the three time points, *F*(2,46) = 19.82, *p* < 0.001, *n* = 24, $\eta_{g}^{2}$ = 0.42. Lower RPQ scores were found at t3 compared to t1, *t*(23) = 3.73, *p* = 0.001, *d* = 0.76, and to t0, *t* (23) = 6.27, *p* < 0.001, *d* = 1.28. Furthermore, RPQ scores were lower at t1 in comparison to t0, *t*(23) = 2.87, *p* = 0.005, *d* = 0.59.

**Table 2:** Mean values, SD, and Ranges of the Mental Health Measures at t0, t1, t3.

	t0			t1			t3		
Scale	*M*	SD	Range	*M*	SD	Range	*M*	SD	Range
CDI score^a^	7.26	3.89	1–17	6.52	3.51	0–15	3.15	2.01	0–10
SDQ score^b^	11.64	4.52	4–22	8.61	3.38	4–17	7.18	2.02	4–11
RPQ score^c^	10.54	6.53	0–24	5.88	5.21	0–19	1.83	1.83	0–6

*^a^*n* = 27; ^b^*n* = 28; ^c^*n* = 24. t0, baseline survey, 20 months before intervention; t1, pre-assessment, 1–4 weeks before intervention; t3, follow-up assessment, 3 months after intervention; CDI, Children’s Depression Inventory; SDQ, Strengths and Difficulties Questionnaire; RPQ, Reactive-Proactive Questionnaire*.

## Discussion

In several low-income countries, high rates of maltreatment and poor caregiving quality in institutional care have been repeatedly shown (Muhamedrahimov et al., 2004; Hermenau et al., 2011; Euser et al., 2014). In response to these high rates of maltreatment, our training approach has been developed. The current study replicated these findings: 87% of the caregivers and 93% of the children reported occurrences of maltreatment in institutional care, demonstrating the need for an intervention focusing on maltreatment prevention (Gavrilovici, 2014; Hermenau et al., 2014). Furthermore, the present study revealed that caregivers showed enhanced stress levels and shared very little quality time with children. These findings resemble previous findings highlighting the poor working conditions of caregivers and the low caregiving quality (Heron and Chakrabarti, 2002; McCall, 2013; Wright et al., 2014). Therefore, the need for a training approach that improves the living and working conditions in institutional care, both for children and caregivers, is extremely high.

Previous training approaches concentrated mainly on the improvement of the caregiving quality and the prevention of neglect (Groark et al., 2005; Rygaard, 2010). The present training approach is one of the few that focuses additionally on the prevention of physical violence and emotional abuse, thereby paying attention to the common use of maltreatment in institutional care and its negative consequences on mental health (Gershoff, 2002; Hermenau et al., 2014; UNICEF, 2014). An important strength of the present study is that the evaluation includes not only the participating caregivers but also the trainers and the children. Particularly through giving the affected children a voice, we can evaluate whether changes in attitude reported by the caregivers are also transferred in caregiving behavior in everyday life.

The first aim was to test the feasibility of our newly developed approach: the trainers showed high satisfaction with the implementation of the units, the caregivers’ participation, comprehension, and motivation. Consistently, caregivers reported being very satisfied with the training. Thus the training was feasible despite the challenging circumstances and the little resources available in this low-income country. We hypothesize that the training approach may also be applicable in other institutions and countries, as the initial conditions were comparable to those found in other studies in low-income countries (Muhamedrahimov et al., 2004; Rygaard, 2010). However, this needs to be tested in future studies.

Since corporal punishment and other harmful discipline methods are very common, socially normed and generally regarded as effective in Tanzania and many other countries (Straus, 2010; Hecker et al., 2014), we expected to be confronted with strong resistance by the trainees to contribute to a change of attitude. However, involving the trainees in creating the change and formulating their own training helped to reduce resistance and promoted engagement in the process. The reflections about the caregivers’ own experiences of maltreatment, the subsequent discussions about consequences of maltreatment for children, and the intensive practicing of effective non-violent caregiving strategies may have facilitated a change of attitude regarding harmful discipline methods and maltreatment as well. This change of attitude can be concluded from the caregiver’s report that they understood the children’s behavior and needs better after the training workshop. A perceived change of behavior may be noted, as the caregivers stated using most of the training aspects in their daily work 3 months after the workshop. Further, the reported improvements in the caregiver–child relationship and child behavior may indicate improvements in the caregiving quality, though we cannot control for other potential influences due to the uncontrolled study design. The training may have influenced the caregivers’ work and subsequently the living and working conditions in the institution. We therefore conclude that the training content was applicable to the caregiver’s daily work – at least short-term – and that they perceived the training to be useful.

Concordantly, Study 2 revealed also that the children reported an improvement in caregiving. They reported that physical maltreatment had decreased 3 months after the training workshop, with a medium effect size. Comparable results were found in a study of Hermenau et al. (2011) after a caregiver training and a ban on violent punishment. Additionally, the children reported more explanations by caregivers when they did something wrong. Similar to Study 1, these alterations may indicate successful changes in the behavior of the caregivers. However, we cannot rule out other potential influences due to the uncontrolled study design. The training approach may be able to contribute to a change from violent punishment as common practice toward an acceptance and implementation of non-violent discipline and caregiving strategies. However, the hypothesized decrease of emotional maltreatment was not found. Possibly, our training approach focused more on physical than emotional maltreatment. Having the detrimental consequences of emotional abuse in mind, future training workshops should place greater emphasis on emotional maltreatment and its detrimental consequences.

In addition, the children’s mental health improved 3 months after the training workshop: depressive symptoms showed a large decline between pre- and follow-up -assessment, and no significant differences between baseline and pre-assessment. Also, internalizing and externalizing problems as well as aggressive behavior decreased significantly. However, the decrease from baseline to pre-assessment limits a clear attribution of the effect to the training. The employment of a counseling psychologist as a result of the baseline study may have impacted the results. However, in accordance with other research, we argue that changes in the caregiving behavior potentially induced by the training may have further contributed to the decline in mental health problems (McCall, 2013). Concordantly, other studies showed both improvements of care quality and reduction of maltreatment to be linked with improved mental health (Hermenau et al., 2011, 2014; McCall, 2013). Accordingly, there is a high need for well-trained caregivers to improve the mental health of burdened, institutionalized children.

The presented training approach extends existing caregiver training programs, like the FairstartGlobal approach (Rygaard, 2010) or the St. Petersburg study (Groark et al., 2005) by also addressing maltreatment in institutional care. It can be easily implemented in low-income countries without the need for additional resources that cannot be provided in the long term. Although no structural changes were implemented, the training workshop provides first hints that it may induce changes in the attitude and behavior of the caregivers. Changing long-standing norms is challenging; in this case especially because the use of corporal punishment and other violent discipline measures is highly prevalent and widely accepted in the Tanzanian society. Despite this, the caregivers participated with high motivation and were open-minded toward the contents of the training workshop. The flexibility of the training approach to adjust to the caregivers’ needs and given conditions made it possible to cope with the challenging circumstances faced in many low-income countries. The training approach revealed a good implementation and further indicated promising results regarding the efficacy of improving the situation for both children and caregivers. To ensure the efficacy of this approach, further research is necessary. The training approach should be tested using controlled designs and validated outcome measures, such as in a randomized cluster trial in a multi-center, multi-country study. As earlier studies found decreasing long-term effects (Taneja et al., 2005; McCall et al., 2014), special focus should be placed on testing the long-term sustainability of positive effects on care quality and maltreatment prevention.

The present study has some important limitations. With the small sample of caregivers and children, we cannot claim that there is an inclusive representation of other institutions in other low-income countries. Furthermore, our uncontrolled study design limits conclusions about causal effects. Potential bias, such as social desirability, can never be completely ruled out for self-report measures. Alternative explanations for the decrease in maltreatment and the improvements in mental health cannot be totally excluded as we also found improvements between baseline and pre-intervention interviews. However, these changes can likely be explained by the employment of a school counselor, assigned to support the children in institutional care, following the baseline interviews. The conduction of the caregiver surveys and the exam encountered challenges stemming from reading and writing skill deficiencies.

## Conclusion

Maltreatment prevention in institutional care in low-income countries is an aspect that has predominantly been neglected in research so far, despite the high rates of maltreatment and its repeatedly reported detrimental effects on the children’s well-being (Gershoff, 2002, 2013; Hermenau et al., 2014). The present study closes this gap and provides an attachment theory-based and practice-oriented training workshop that is feasible in challenging circumstances. It further provides first hints that it may contribute to a decrease in maltreatment, mental health problems, and aggression in institutionalized children. Thus, the study on hand suggests that our training approach for caregivers in institutional care may lead to changes in attitude and behavior of caregivers, thereby it may contribute to improve the well-being of children living in institutional care. The promising results of the present study call for further research to test the efficacy and sustainability of this training approach.

## Author Contributions

KH and TH conceived and designed the study, drafted the training manual, participated in data collection, guided data analysis and interpretation, and drafted the manuscript. TH also conducted the training workshop. EK drafted the manuscript, participated in data collection, analyzed and interpreted the data and supported the training workshop. GM participated in data collection, conducted the training workshop, and critically revised the manuscript prior to submission.

## Conflict of Interest Statement

The authors declare that the research was conducted in the absence of any commercial or financial relationships that could be construed as a potential conflict of interest.
