# Supplementary material for: Improving care quality and preventing maltreatment in institutional care – a feasibility study with caregivers
Source: Front Psychol. 2015 Jul 14;6:937. doi: 10.3389/fpsyg.2015.00937 (PMC4501176; doi:10.3389/fpsyg.2015.00937)
Supplement: Supplementary file 1 [file Table_1.DOCX]

Suppl. Table 1.

*Caregiver Surveys at t1, t2, t3*

| **Sociodemographic information** | | | | | **Time point** |
| --- | --- | --- | --- | --- | --- |
| Date of birth: | | | | | t1, t2, t3 |
| Sex: ⬜ female ⬜ male | | | | | t3 |
| How many years did you go to school? | | | | | t1 |
| What is the name of your working place? | | | | | t1 |
| How long do you work as a caregiver? ______ years ______ months | | | | | t1 |
| Did you receive any training in the work with children?  ⬜ yes ⬜ no | | | | | t1 |
| How old are the children you work with?  From ______ years to ______ years | | | | | t1 |
| What are your tasks in the institution? | | | | | t1 |
| **Demand** | | | | | t1 |
| How do you rate your current work load?  0  0  10  very low work load  very high work load | | | | | t1 |
| How many hours do you work in a week? | | | | | t1 |
| CBI: work-related burnout | | | | | t1 |
| **Motivation** | | | | |  |
| How motivated are you to participate in this workshop?  0  0  10  not at all  very much | | | | | t1 |
| How much do you think this workshop will help you in your daily work?  0  0  10  not at all  very much | | | | | t1 |
| **Feasibility of the training** | | | | |  |
| How satisfied were you with the workshop? | | | | | t2 |
| ⬜  excellent | ⬜  good | ⬜  satisfying | ⬜  unsatisfying | ⬜  not adequate |  |
| Would you recommend this workshop to others?  ⬜ yes ⬜ no | | | | | t2 |
| If you would have to contribute money for the workshop. How much money would you be willing to contribute for each day? | | | | | t2 |
| **Efficacy** | | | | |  |
| How did the workshop influence your understanding of the children? | | | | | t2 |
| How would your work load change by implementing your new knowledge in your daily work? | | | | | t2 |
| ⬜ much lower work load | ⬜ a bit lower work load | ⬜ same work load | ⬜ a bit higher work load | ⬜ much higher work load |  |
| How many aspects of the workshop can you use in your daily work? | | | | | t3 |
| ⬜ no aspects | ⬜ a few aspects | ⬜ half of the aspects | ⬜ most of the aspects | ⬜ all aspects |  |
| Which aspects of the workshop do you use in your daily work? | | | | | t3 |
| Have there been any changes in your workplace since the workshop?  ⬜ yes ⬜ no  If yes, which changes have there been and how do the changes affect your own work? | | | | | t3 |
| Did the relationship to the children you care for change because of the workshop? | | | | | t3 |
| ⬜ much better relationship | ⬜ better relationship | ⬜ no change in the relationship | ⬜ worse relationship | ⬜ much worse relationship |  |
| Do you see a change in the behavior of the children you care for because of you acting differently? | | | | | t3 |
| ⬜ much better behavior | ⬜ better behavior | ⬜ no change in behavior | ⬜ worse behavior | ⬜ much worse behavior |  |
| **Exam** | | | | |  |
| From what do children learn more?  ⬜ From what caregivers say?  ⬜ From what caregivers do? | | | | | t2 |
| Name 3 things that help to maintain the good behavior of a child. | | | | | t2 |
| Name 3 things that help to change the bad behavior of a child. | | | | | t2 |

| Why is a good relationship between caregiver and child important?   1. A good relationship between caregiver and child lays the base for a healthy development. 2. Because of a good relationship between caregiver and child the child will never show misbehavior.   Because of a good relationship between caregiver and child the child will always show good behavior. | t2 |
| --- | --- |
| How should caregivers behave to be a good role model? | t2 |
| Name 3 things that are important when you talk to a child. | t2 |
| Collect the right answer:  Which consequences does corporal punishment have?   1. The child fears the person who uses harmful punishment. 2. The child changes the behavior and behaves well.   The child learns respect. | t2 |
| Collect the right answer:  Why do children behave aggressive?   1. They like to be aggressive and to molest others. 2. They act aggressive because they experienced violence in the family.   They want to bother the caregiver. | t2 |
| Name 3 strategies to help a child to overcome bedwetting. | t2 |
| Name 3 routines or rituals which can help the children and you to structure the day. | t2 |
| Imagine the following situation: Your child is very angry, hits other children und takes away their toys. What will you do? | t2 |

*Notes.* t1 = pre-survey, immediately before the intervention; t2 = post-survey, immediately after the intervention; t3 = follow-up survey, 3 months after the intervention.
