# Supplementary material for: Improving care quality and preventing maltreatment in institutional care – a feasibility study with caregivers
Source: Front Psychol. 2015 Jul 14;6:937. doi: 10.3389/fpsyg.2015.00937 (PMC4501176; doi:10.3389/fpsyg.2015.00937)
Supplement: Supplementary file 2 [file Table_2.DOCX]

# Suppl. Table 2.

*Evaluation of the Training Sessions*

| Unit: | | | | | | |
| --- | --- | --- | --- | --- | --- | --- |
| Instructor: | | | | | | |
| Method: | | | | | | |
| ⬜ Lecture | ⬜ Discussion | ⬜ Small group | | ⬜ Role play | | ⬜ other: |
| Duration: | | | | | | |
| Time management (e.g. enough time for questions) | | | | | | |
| ⬜ excellent | ⬜ good | ⬜ satisfying | ⬜ unsatisfying | | ⬜ not adequate | |
| Participation | | | | | | |
| ⬜ excellent | ⬜ good | ⬜ satisfying | ⬜ unsatisfying | | ⬜ not adequate | |
| Participants’ comprehension of the topic | | | | | | |
| ⬜ excellent | ⬜ good | ⬜ satisfying | ⬜ unsatisfying | | ⬜ not adequate | |
| Motivation of the participants | | | | | | |
| ⬜ excellent | ⬜ good | ⬜ satisfying | ⬜ unsatisfying | | ⬜ not adequate | |
| Overall impression of the feasibility | | | | | | |
| ⬜ excellent | ⬜ good | ⬜ satisfying | ⬜ unsatisfying | | ⬜ not adequate | |
| Difficulties: | | | | | | |
| Ideas for improvement: | | | | | | |
